# Supplementary figures and images for: Impact of short-read sequencing on the misassembly of a plant genome
Source: BMC Genomics. 2021 Feb 2;22:99. doi: 10.1186/s12864-021-07397-5 (PMC7852129; doi:10.1186/s12864-021-07397-5)

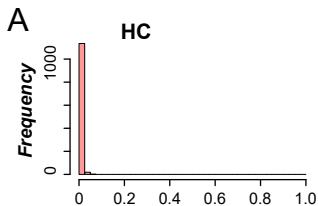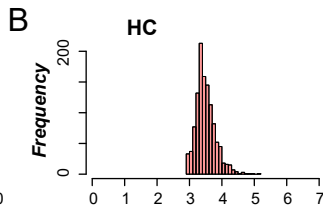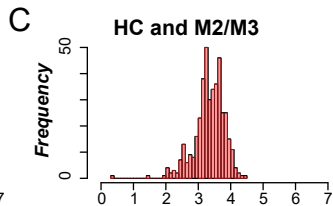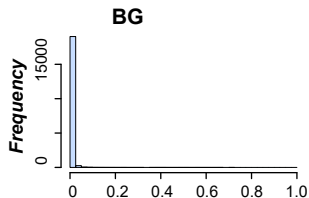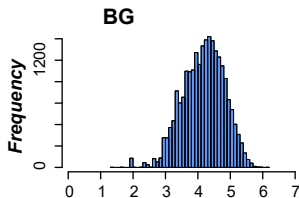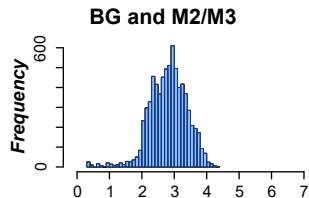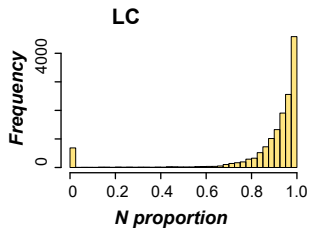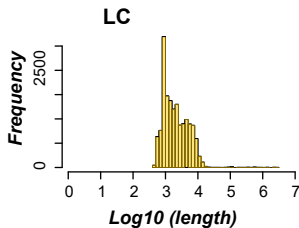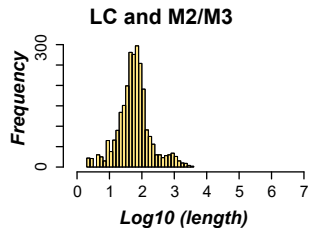

Supplement: Supplementary file 1 — Additional file 1: Figure S1. Properties of HC/LC/BG regions with high confidence. a Proportion of Ns in HC/LC/BG regions. b Length distribution of HC/LC/BG regions. c Length distribution of overlapped regions between HC/LC/BG and M2/M3 regions. [file 12864_2021_7397_MOESM1_ESM.pdf]

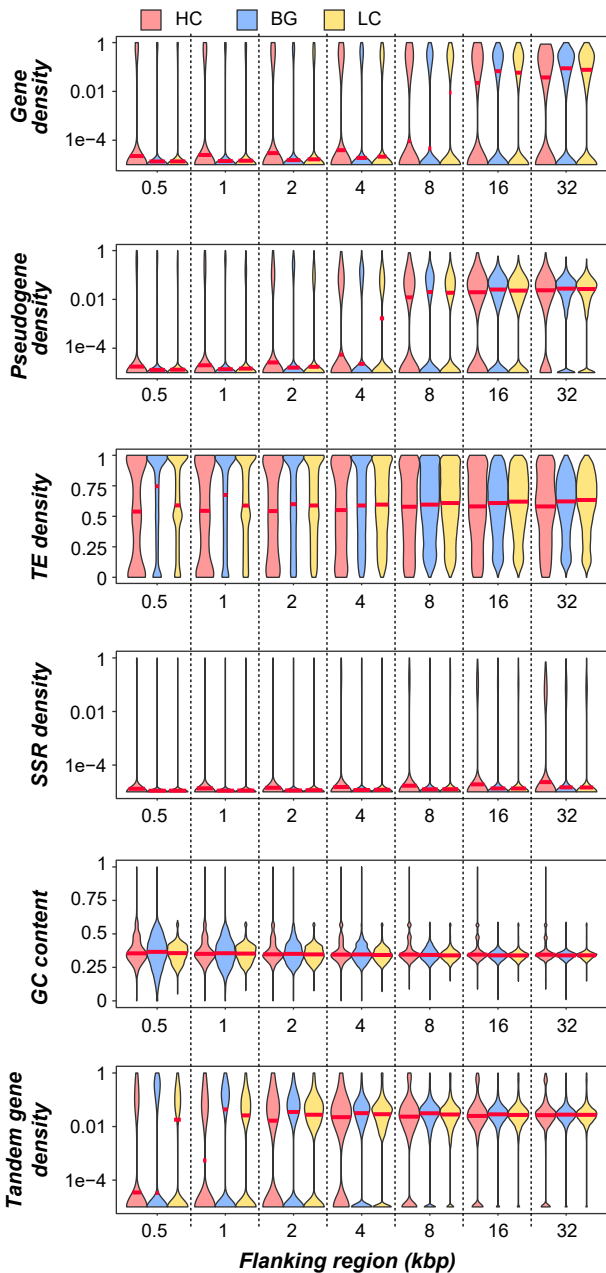

Supplement: Supplementary file 2 — Additional file 2: Figure S2. Genomic feature distributions in flanking regions of HC/LC/BG regions of different length (0.5 ~ 32Kb). Violin plots showing distributions of GC content, and densities of genes, tandemly duplicated genes, pseudogenes, transposable element and SSRs in HC, BG, and LC regions. Red line indicates median value. [file 12864_2021_7397_MOESM2_ESM.pdf]

**Frequency**

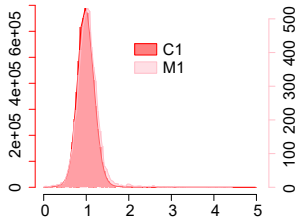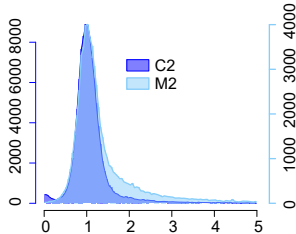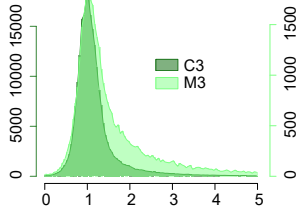

Supplement: Supplementary file 3 — Additional file 3: Figure S3. RD distribution of 100 bp BG bins overlapped with aligned region categories. For each plot, left Y-axis: number of BG bins in correctly assembled category (C1, C2 or C3); right Y-axis: number of BG bins in mis-assembled category (M1, M2 or M3). The categories are defined in Fig. 5a. [file 12864_2021_7397_MOESM3_ESM.pdf]

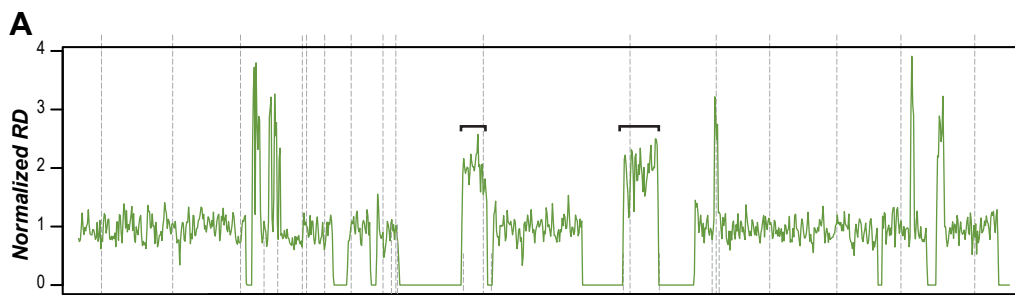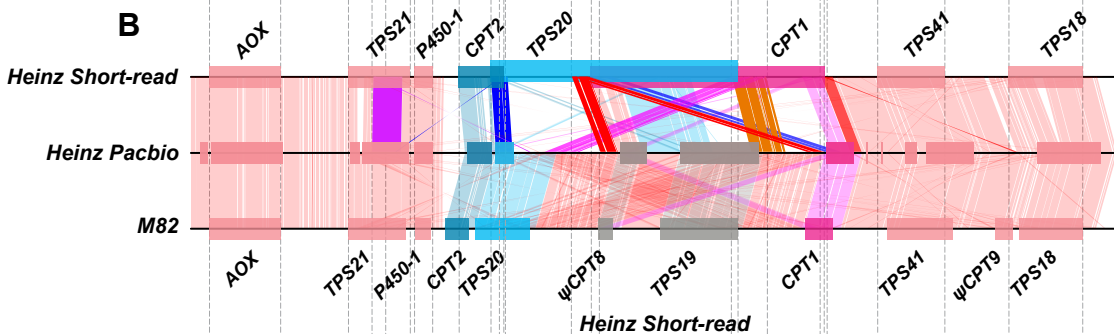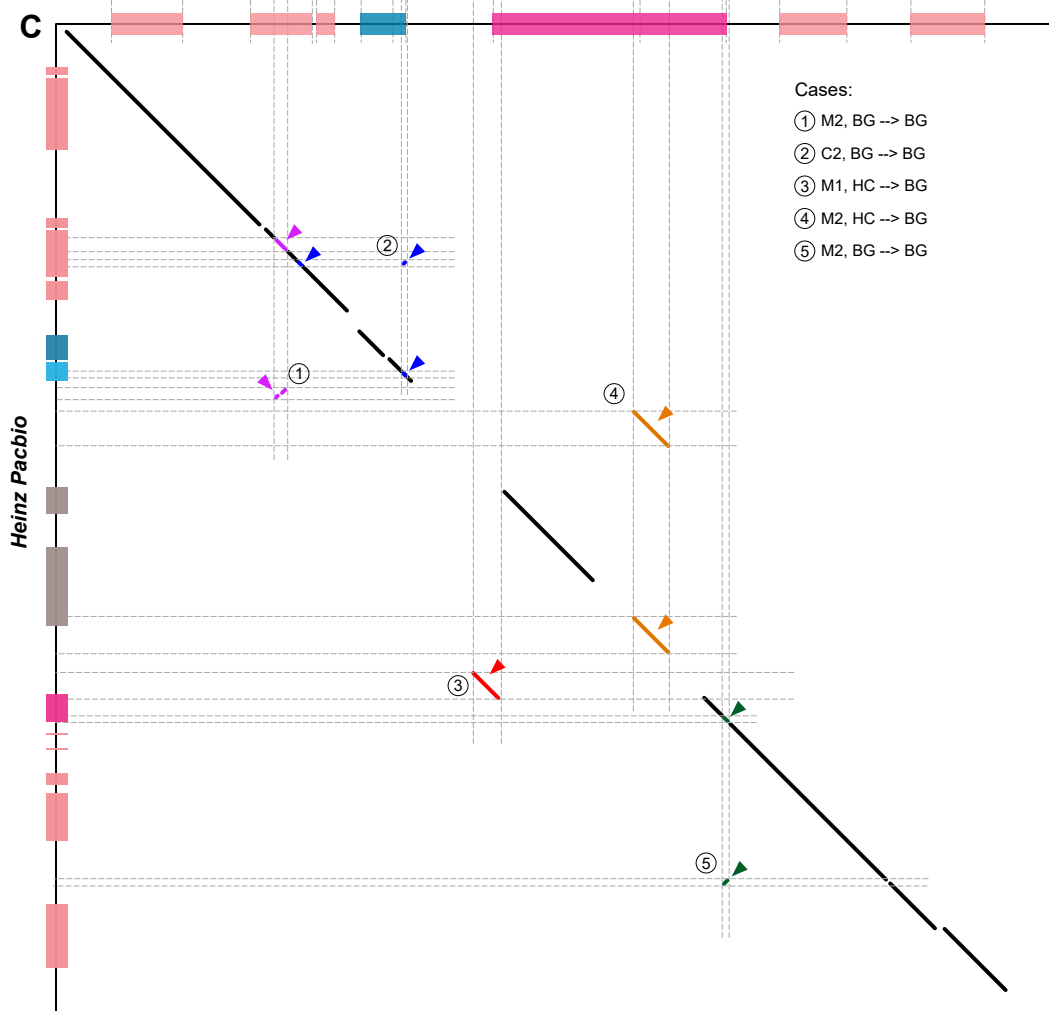

Supplement: Supplementary file 4 — Additional file 4: Figure S4. Example mis-assembled HC regions. a Normalized RD in a region containing an SM gene cluster on Chromosome 8. Normalized RD was calculated for each 100 bp bin. Two black brackets indicate identified HC regions, with RD around 2. Valley regions with RD = 0 indicate gaps in genome assembly filled up with Ns. b Syntenic alignments (colored regions between black lines) and gene annotation (colored boxes) of the same region in (a) between short-read and long-read assemblies of Heinz and the PCR-validated sequence in M82. Corresponding positions in (a) and (b) were delineated with grey dashed lines. Colored regions: BLASTn matches between Heinz and M82, with E-value <1e-20 and alignment length > 50 bp. AOX: alcohol oxidase; TPS: terpene synthases; P450: cytochrome P450; CPT: cis-prenyl transferase; ψ: pseudogene. c Dotplot of the region in (a) between Short- and Long-read assemblies. Five cases were indicated using arrow heads, and all these five short regions were identified to be in BG regions in long-read based assembly (➔ BG). [file 12864_2021_7397_MOESM4_ESM.pdf]

HC\_C2/C3  
HC\_M2/M3

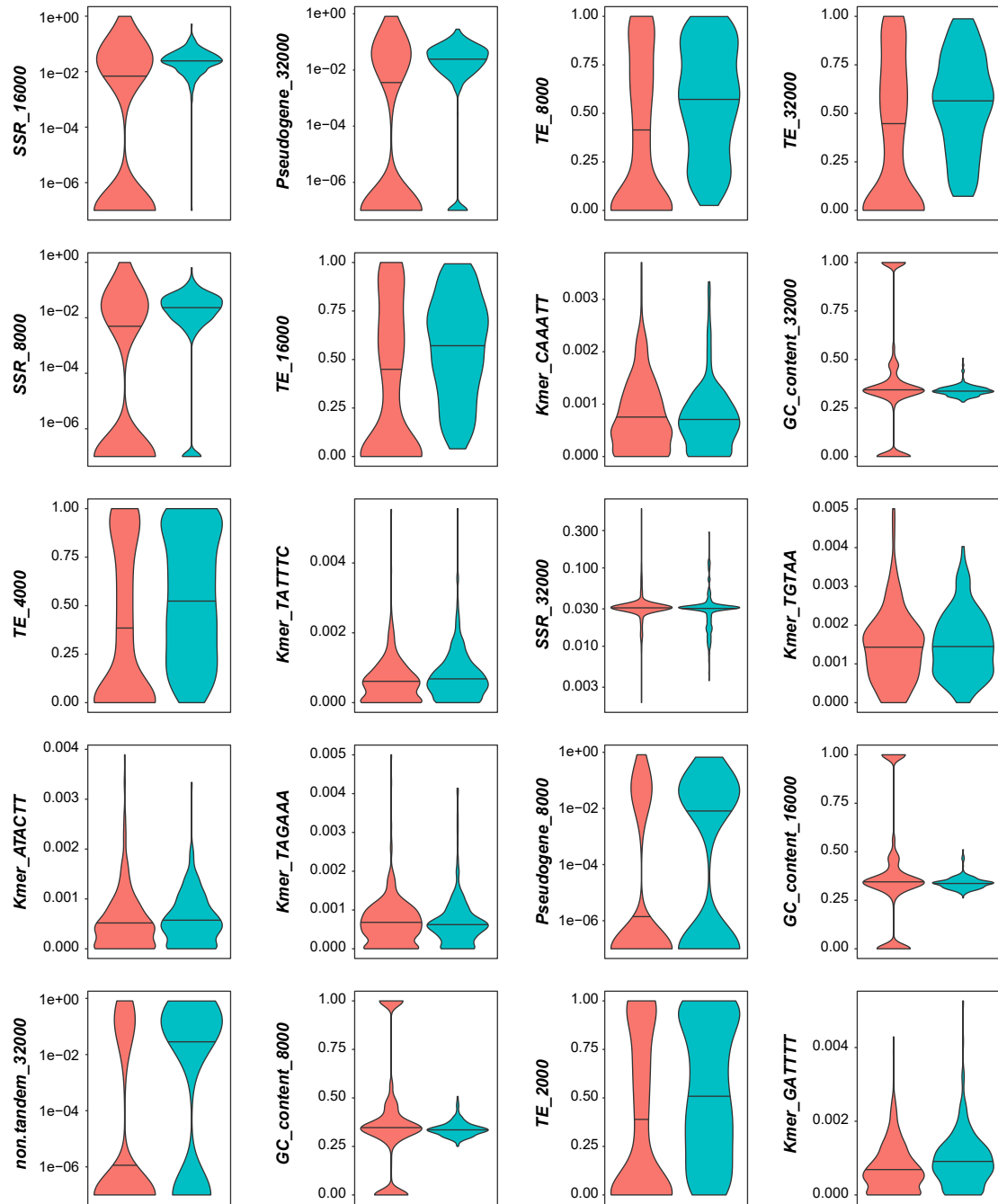

Supplement: Supplementary file 5 — Additional file 5: Figure S5. Important features in model distinguishing HC_M2/M3 and HC_C2/C3. Violin plots show distributions of each features or GC contents within regions or in flanking regions. Lines within violin plots indicate median values. [file 12864_2021_7397_MOESM5_ESM.pdf]

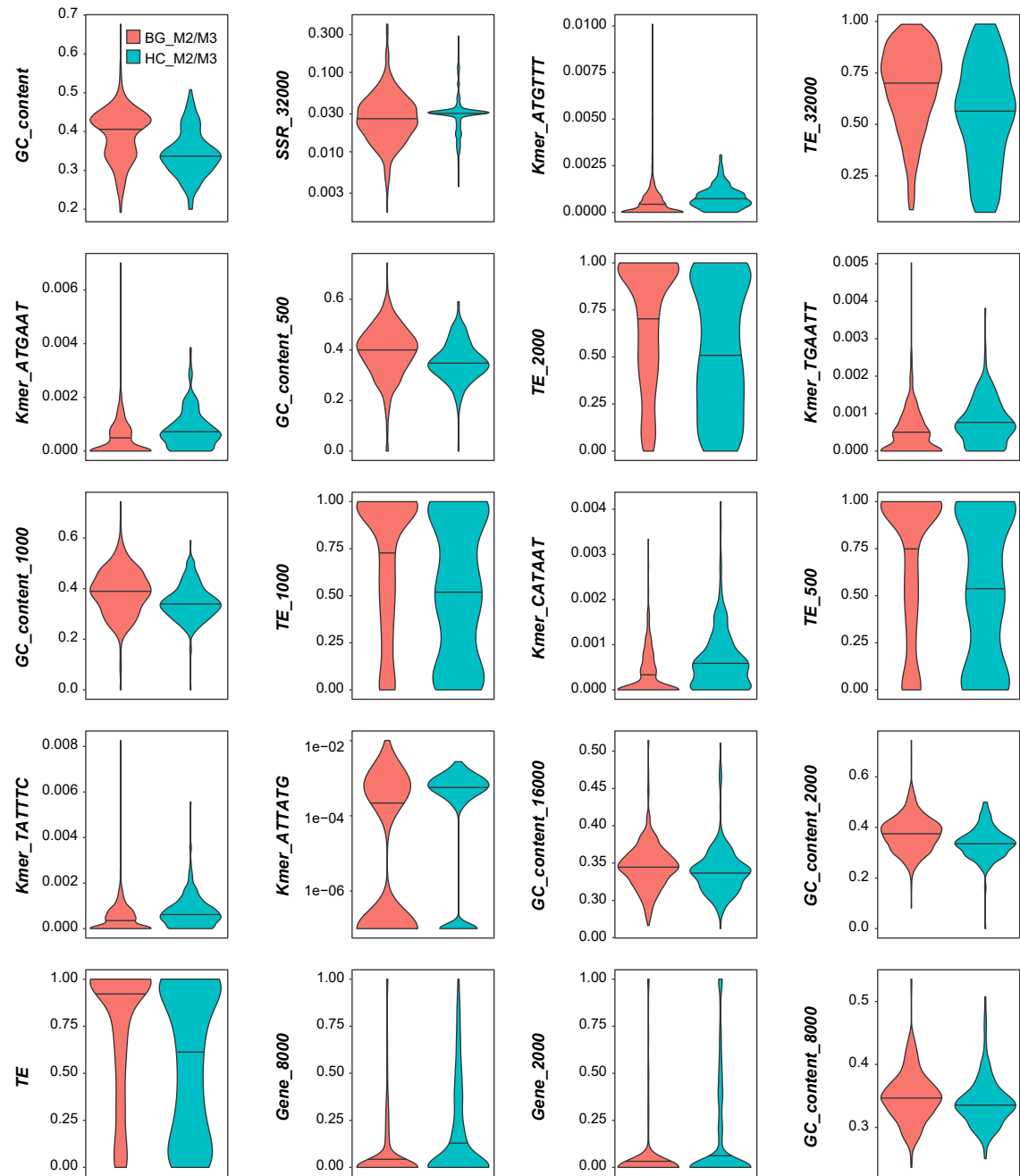

Supplement: Supplementary file 6 — Additional file 6: Figure S6. Distributions of important features in model distinguishing HC_M2/M3 and BG_M2/M3. Violin plots show distributions of each features or GC contents within regions or in flanking regions. Lines within violin plots indicate median values. [file 12864_2021_7397_MOESM6_ESM.pdf]

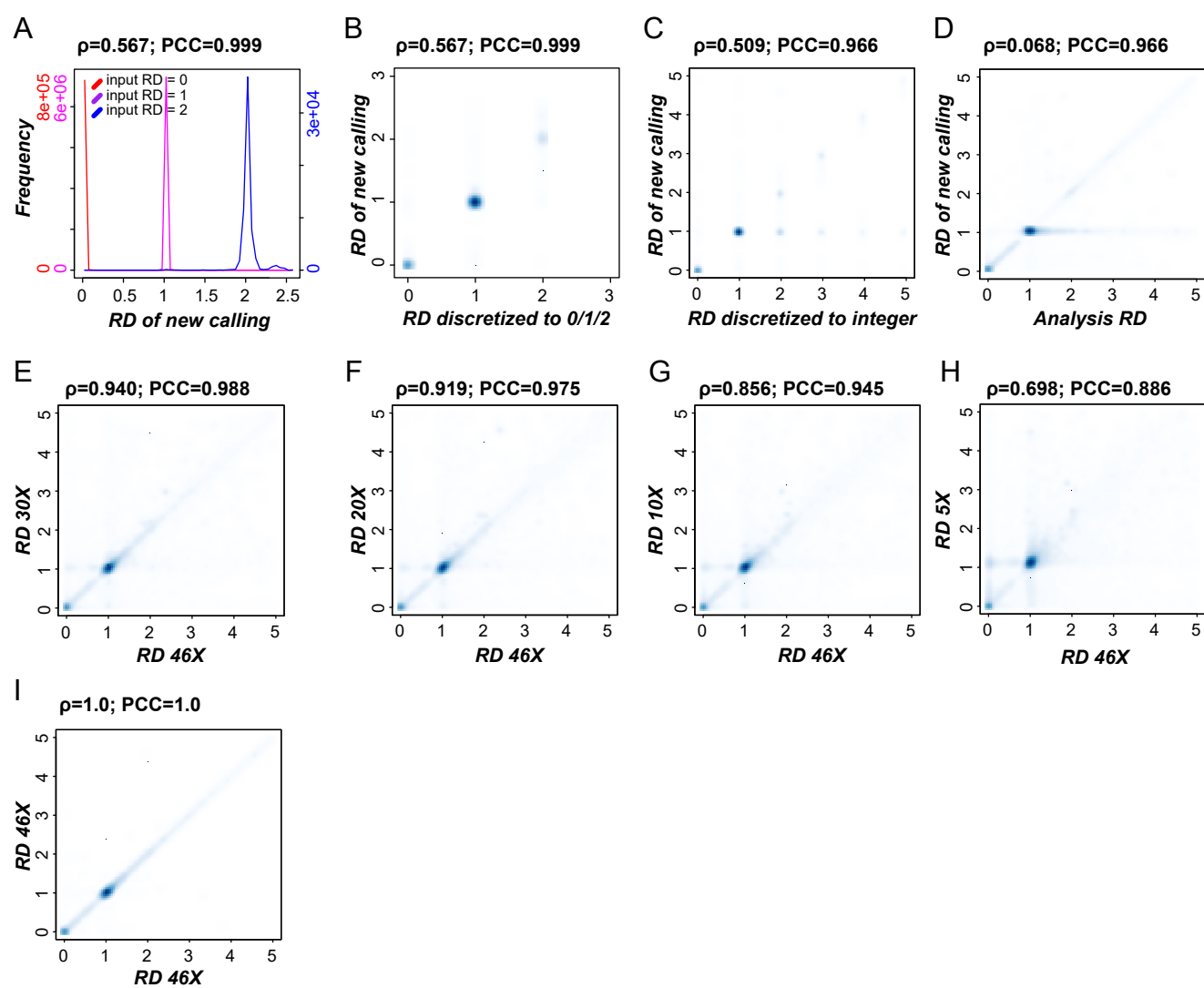

Supplement: Supplementary file 7 — Additional file 7: Figure S7. Sensitivity and accuracy of CNVnator in RD value calculation, and impact of genome coverages on RD values. a RD distribution of new CNVnator runs by mapping re-sampled reads from the tomato genome based on simulated input RD, where the only possible RD values were 0 (LC), 1 (BG), or 2 (HC). b-d Correlation between known, simulated input RDs and new RD values from new CNVnator run using the resampled reads. In (b), the simulated RD values were generated as in (a). In (c), the analysis RD values (those generated with CNVator by mapping dataset2 reads on to the tomato genome, see Methods) were first discretized (rounded) to their closest integers, then the rounded RD values were used for resampling reads for determining new RD values. In (d), the analysis RD values were directly used for resampling reads for determining new RD values. e-i Correlation between RD values using all reads (46X coverage) and RD values using subsets of reads at variable coverage: (e) 30X, (f) 20X, (g) 10X, (h) 5X, (i) 46X. [file 12864_2021_7397_MOESM7_ESM.pdf]
