# Supplementary material for: Impact of short-read sequencing on the misassembly of a plant genome
Source: BMC Genomics. 2021 Feb 2;22:99. doi: 10.1186/s12864-021-07397-5 (PMC7852129; doi:10.1186/s12864-021-07397-5)
Supplement: Supplementary file 8 — Additional file 8: Figure S8. F1 of HC/LC/BG region calling. a-d CNVnator runs were conducted using resampled reads based on different starting RD values as in Fig. S7 using read dataset 2. As in Fig. S7, the simulated RD values include: (a) possible RD values of only 0 (LC), 1 (BG), or 2 (HC); (b) analysis RD values discretized/rounded to integers; (c) analysis RD without discretization; (d) analysis RD without discretization but down-sampled to 30X (note the y-axis range is much smaller compared to (a-c)). Each dot indicates an F1 value (y-axis) at a given q-value threshold (x-axis), where F1 was calculated using: (1) numbers of nucleotides in overlapping regions between the HC/LC region designations based on the analysis RD and new HC/LC regions determined using resampled reads (True Positive), (2) numbers of nucleotides in true HC/LC regions but determined as BG regions in new run (False Negative), and (3) numbers of nucleotides in true BG regions but determined as HC/LC regions in new run (False Positive, see Methods). Orange line: LOESS fitted curve. e-h Same as (a-d) except that the F1 was determined based on numbers of regions as opposed to numbers of nucleotides. [file 12864_2021_7397_MOESM8_ESM.pdf]

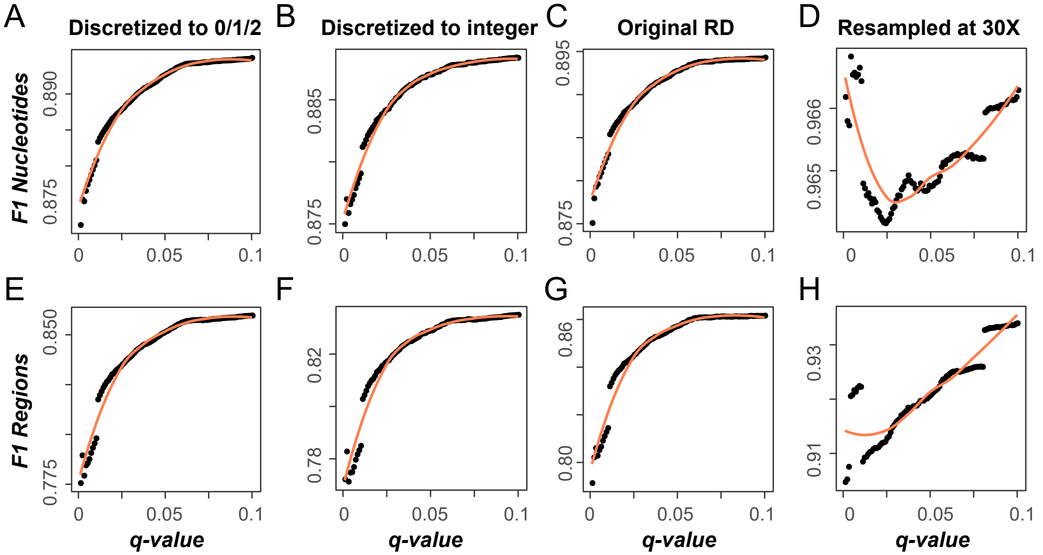
A
Discretized to 0/1/2
B
Discretized to integer
C
Original RD
D
Resampled at 30X
F1 Nucleotides
0.875
0.890
0.885
0.895
0.965
0.966
0
0.05
0.1
E
F
G
H
F1 Regions
0.775
0.850
0.78
0.82
0.80
0.86
0.91
0.93
q-value
